# New lower jaw and teeth referred to *Maxakalisaurus topai* (Titanosauria: Aeolosaurini) and their implications for the phylogeny of titanosaurid sauropods

**DOI:** 10.7717/peerj.2054

**Published:** 2016-06-08

**Authors:** Marco A.G. França, Júlio C. de A. Marsola, Douglas Riff, Annie S. Hsiou, Max C. Langer

**Affiliations:** 1 Laboratório de Paleontologia e Evolução de Petrolina, Colegiado de Ciências Biológicas, Universidade Federal do Vale do São FranciscoPetrolina,Pernambuco,Brazil; 2 Laboratório de Paleontologia de Ribeirão Preto, FFCLRP, Universidade de São Paulo, Universidade de São Paulo,Ribeirão Preto,São Paulo,Brazil; 3 Laboratório de Paleontologia, Instituto de Biologia, Universidade Federal de UberlândiaUberlândia, Minas Gerais,Brazil

**Keywords:** Dinosauria, Titanosauria, Cretaceous, Phylogeny, Evolution, Bauru Group, Adamantina Formation

## Abstract

Sauropod dinosaurs compose a diversified, well known, and worldwide distributed clade, with a stereotyped body plan: deep trunk, elongated neck and tail, columnar limbs and very small skull. In Brazil, the group is represented by ten formally described Cretaceous species, mostly titanosaurs. This is the case of *Maxakalisaurus topai*, known based on an incomplete and disarticulated skeleton, unearthed from deposits of the Adamantina Formation in Minas Gerais. Here, we report a partial right dentary, including five isolated teeth, collected from the same site as the type-series of *M. topai* and tentatively referred to that taxon. The bone is gently curved medially, the functional teeth are set on an anterolingual position, and two replacement teeth are seen per alveoli. New morphological data gathered from that specimen was employed to conduct a comprehensive phylogenetic analysis of Titanosauria (with 42 taxa and 253 characters), based on previous studies. The Aeolosaurini clade was recovered, with *Gondwanatitan* and *Aelosaurus* as sister taxa, and *Maxakalisaurus*, *Panamericansaurus*, and *Rinconsaurus* forming a basal polytomy.

## Introduction

Sauropodomorpha is a clade of herbivorous dinosaurs that originated during the Late Triassic and were prevalent, both in diversity and biomass, in terrestrial biomes during the Middle-Late Mesozoic, with at least 175 valid taxa currently known (Young, 1951; Dodson, 1990; Galton, 1986; Langer et al., 1999; Barret & Upchurch, 2005; Martinez & Alcober, 2009; Mannion et al., 2011; Novas et al., 2011). them, Sauropoda is not only the most diverse clade, but the second most representative dinosaurian group - c. 18% of the non-avian dinosaur diversity (Curry Rogers & Wilson, 2005). The sauropod body plan is unique among terrestrial tetrapods, with a short and deep trunk combined to a very small skull and a very long neck and tail, and massive and columnar limbs that only enables the quadrupedal, graviportal locomotion (Sander et al., 2011; Bates et al., 2016). The earliest diverging forms, such as *Vulcanodon*, *Shunosaurus*, and *Jobaria*, are arranged on successively closer positions to Neosauropoda, which includes Diplodocoidea and Macronaria (Wilson, 2002). Within Macronaria, Titanosauria represents the most speciose clade, corresponding to one of the most abundant Cretaceous dinosaur groups, particularly successful in Gondwana, mainly in the South America mainland (Salgado, Coria, & Calvo, 1997; Wilson, 2002). The group includes early splits (e.g., *Phuwiangosaurus*) and the Lithostrotia lineage, also including early splits and clades like Nemegtosauridae, Saltasaurinae, Opisthocoelicaudinae, and Aeolosaurini (Apesteguia, 2004; Wilson & Upchurch, 2003; Upchurch, Barrett & Dodson, 2004; Santucci & Arruda-Campos, 2011).

**Figure 1:**
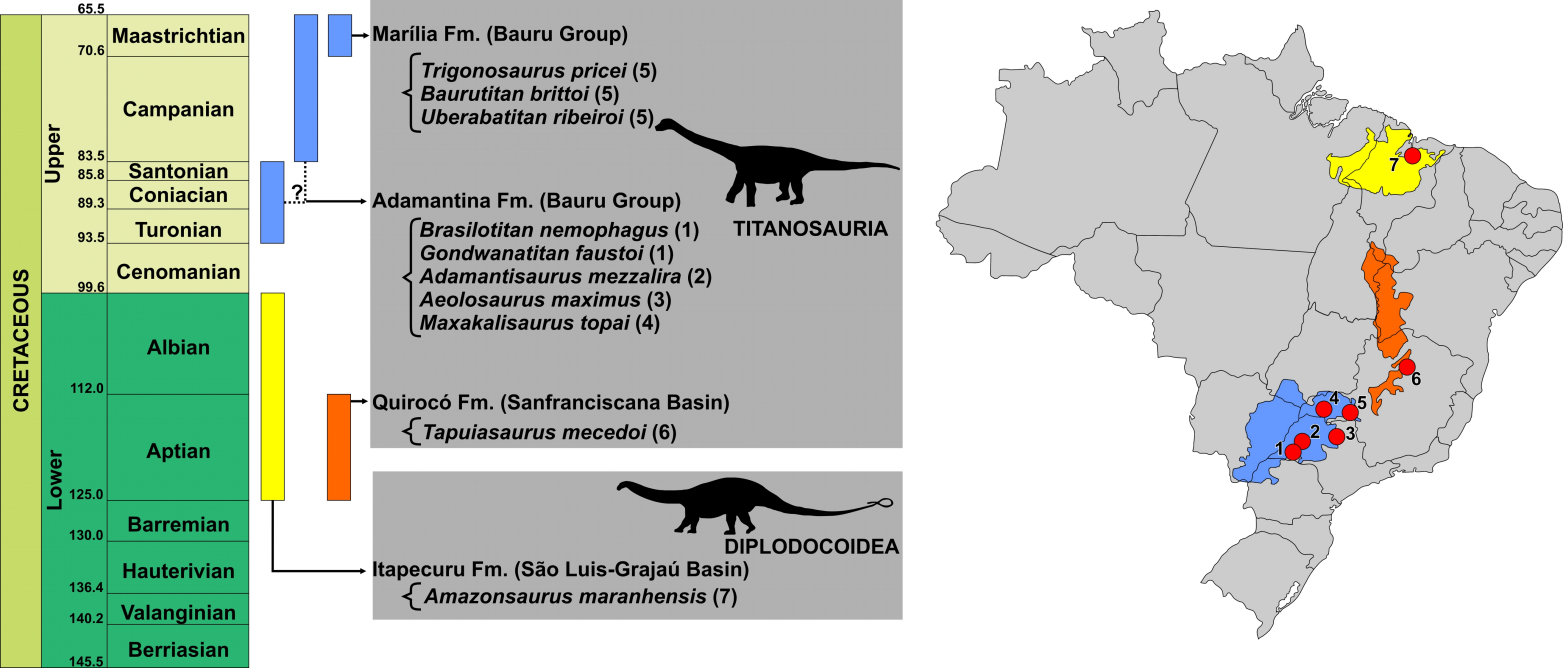
Temporal and geographic distribution of Brazilian Cretaceous Sauropods. Blue: Bauru Basin - Adamantina (1, Presidente Prudente and Alvarez Machado, São Paulo; 2, Flórida Paulista, São Paulo; 3, Monte Alto, São Paulo; 4, Campina Verde, Minas Gerais) and Marília (5, Peirópolis, Minas Gerais) formations. Orange: Sanfranciscana Basin - Quirocó Formation (6, Coração de Jesus, Minas Gerais). Yellow: São Luis-Grajaú Basin - Itapecuru Formation (7, Itapecuru-Mirim, Maranhão).

Sauropods are currently represented in Brazil by ten formally proposed Cretaceous taxa (Fig. 1), all within the Titanosauria clade, except for the diplodocoid *Amazonsaurus maranhensis* (Carvalho, Avilla & Salgado, 2003). Described by Kellner et al. (2006) from deposits of the Adamantina Formation, in Minas Gerais, M*axakalisaurus topai* corresponds to one of those titanosaurs, and seems closely related to the Aelosaurini group (Santucci & Arruda-Campos, 2011). The taxon is based on a disarticulated bone assemblage, including an incomplete maxilla, several vertebrae (twelve cervical, seven dorsal, one sacral, and six caudal elements), at least three chevrons, one osteoderm, as well as some scapular and pelvic elements such as scapulae, sternal plates, humeri, metacarpals, ischium, and fibula (Kellner et al., 2006). Recently, new field incursions to the type-locality of *M. topai* (Kellner et al., 2006; Batezelli, 2015) unearthed the additional specimen here analyzed. The new elements provide data to better understand the morphology and phylogeny of titanosaurids.

## Materials and Methods

### Material

The new fossil material was collected from a fine to medium grained reddish sandstone of the Adamantina Formation (Bauru Group, Upper Cretaceous), at the type-locality of *Maxakalisaurus topai*, located about 45 km west of Prata (Minas Gerais), at the Prata-Campina Verde road. The material includes an incomplete right dentary and isolated teeth found on a subparallel arrangement, housed at the Zoological Collection of INBIO/UFU, under numbers MBC-42-PV and MBC-38-PV, respectively. The dentary shows external crackled surface and some longitudinal fractures, an indicative of prolonged subaerial exposure, as also seen in the type-series. The assemblage, collected in an area of 40 m^2^, consists of supposedly associated and autochthonous remains restricted to a single horizon (Kellner et al., 2006). It also includes isolated turtle shell fragments, crocodylomorph and theropod teeth, and the sauropod bones were all associated to *M. topai*. The anterior maxillary fragment of the holotype measures approximately 5 cm in length, and bears five alveoli with functional and replacement teeth. The dentary (MBC-42-PV) described here is 8.2 cm in length, close to the size expected for that of the holotype. In addition, the pencil-like teeth of holotypic maxilla is very similar in length and shape to isolated teeth of MBC-38-PV. Put together, these data allow associating MBC-42-PV and MBC-38-PV to *M. topai*.

### Phylogenetic analysis

In order to conduct the phylogenetic study, two recent analyses for Sauropoda (Santucci & Arruda-Campos, 2011; Zaher et al., 2011) were reviewed. Both are based on the original study of Wilson (2002) and relatively well sampled for Brazilian sauropods. The resulting matrix contains 253 characters: 234 originally proposed by Wilson (2002), seven added by Santucci & Arruda-Campos (2011) and twelve by Zaher et al. (2011). Likewise, the 42 terminal taxa correspond to the 29 taxa used in Wilson (2002), plus the nine added by Santucci & Arruda-Campos (2011) and the four added by Zaher et al. (2011). Two heuristic searches were carried out (using Tree Bisection and Reconnection, 10,000 replicates, and hold of 10) on TNT (Goloboff, Farris & Nixon, 2008), with characters 8, 37, 64, 66, and 198 ordered, as in the original analysis of Wilson (2002). In the first analysis, the scoring for *M. topai* was based only on previous works, whereas the second includes the new data gathered here from the referred dentary and teeth. In addition, Retention and Consistency Indices were obtained for the two analysis, using STATS script on TNT (Goloboff, Farris & Nixon, 2008). Branch-support were evaluated by Bremer support (Bremer, 1994), using Bremer Script on TNT (Goloboff, Farris & Nixon, 2008), and Boostrap analysis (Felsenstein, 1985) also implemented on TNT, with 3,000 replicates for search suboptimal tress in steps with tree bisection-reconnection branch swapping.

## Systematic Paleontology

**Table:** 

SAUROPODOMORPHA Huene, 1932
SAUROPODA Marsh, 1878
MACRONARIA Wilson & Sereno, 1998
TITANOSAURIA Bonaparte & Coria, 1993
LITHOSTROTIA Wilson & Upchurch, 2003
AEOLOSAURINI Franco-Rosas et al., 2004
*Maxakalisaurus topai*Kellner et al., 2006

*Holotype:* Partial skeleton composed of an incomplete right maxilla (with teeth), the remains of 12 cervical vertebrae (including several cervical ribs), part of seven dorsals (and ribs), one sacral neural spine, one sacral centrum, six caudals, several hemal arches, part of both scapulae, both sternal plates, the distal portion of a left ischium, both humeri, the second and forth right metacarpals, incomplete fibula, one osteoderm, and several unidentified bones. This specimen (MN 5013-V) is housed at the Museu Nacional of the Universidade Federal do Rio de Janeiro, Rio de Janeiro, Brazil.

*Referred materials:* MN 7048-V, distal end of a right scapula, and MN 7049-V and MN7050-V, two sternal plates, housed at the Museu Nacional of the Universidade Federal do Rio de Janeiro; MBC-42-PV, incomplete right dentary, and MBC-38-PV, isolated teeth found on a subparallel arrangement, housed at the Zoological Collection of INBIO/UFU.

*Type locality and horizon:* 45 km west of the Prata town, at the Prata – Campina Verde road, in a region called Serra da Boa Vista, Minas Gerais State, Brazil; Adamantina Formation, Bauru Group, Upper Cretaceous (Kellner et al., 2006; Batezelli, 2015).

*Emended diagnosis:* Titanosaur dinosaur characterized by the following combination of features: Meckelian channel not enters on symphysis area on dentary; tooth row in U-shaped; teeth with high-angled planar facets and suboval in cross-section; two replacement teeth per alveolus; tail composed of anterior and midposterior caudal vertebrae with the anterior (and posterior) surface of the centrum dorsoventrally compressed; midposterior caudal vertebrae with the lateral surface of the centrum strongly concave (spool-shaped); dorsal margin of neural spine in midposterior caudal vertebrae inclined anteriorly; presence of at least one midposterior caudal with biconvex centrum; metacarpal IV about 12% shorter than metacarpal II; sacral centrum with keel-shaped ventral surface.

**Figure 2:**
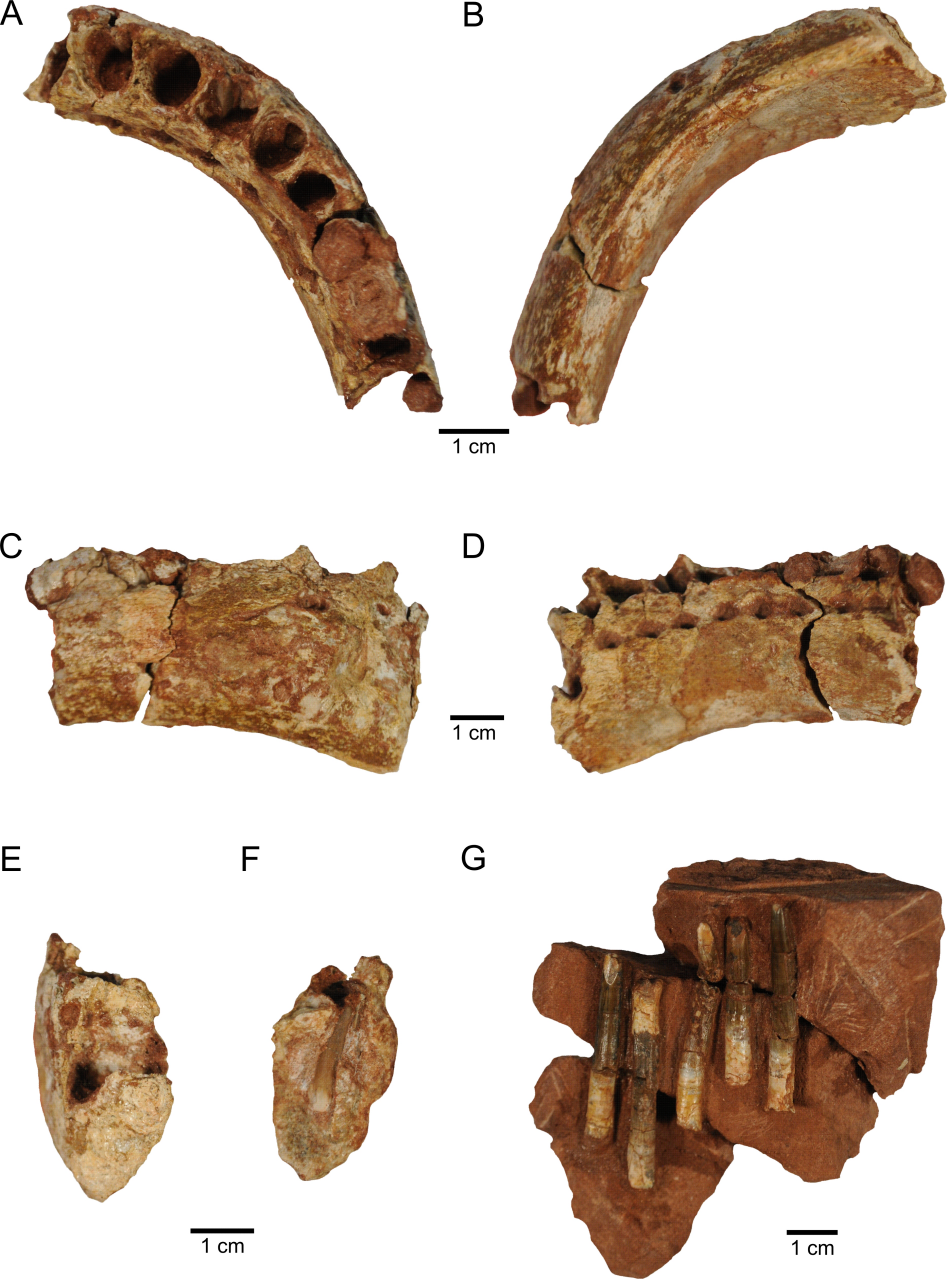
New material of *M. topai*. (A–F), MBC-42-PV, right dentary in dorsal (A), ventral (B), lingual (C), labial (D), and symphyseal (E) views; (F), cross section at the level of the seventh alveolus, showing one replacement tooth. (G), MBC-38-PV, functional teeth as found in the bearing rock.

## Results and Discussion

### Description

The dentary, MBC-42-PV (Fig. 2), corresponds to the anterior part of the right element, including the symphyseal region and ten alveoli. The fragment measures 8.2 cm in anteroposterior length, with 3.6 and 4.4 cm of minimal and maximal dorsoventral depth respectively. This appears to encompass the almost entire alveolar region, as basal titanosaurs have up to 15 dentary alveoli, e.g., *Malawisaurus* (Gomani, 2015), and this number varies from 11 to 13 in Lithostrotia, as seen in *Rapetosaurus* and *Nemegtosaurus* respectively (Curry Rogers & Forster, 2004; Nowinski, 1971). In dorsal/ventral view, the dentary gently curves anteromedially, forming a 45 degrees angle between its anterior and posteriormosts portions. The regions of alveoli 1–3 and 7–10 are straighter in dorsal/ventral view, whereas that of alveoli 4–6 holds the curvature. This morphology is similar to that seen in *Brachiosaurus* (Janensch, 1935–36), *Euhelopus* (Poropat & Kear, 2013),and *Tapuiasaurus* (Zaher et al., 2011), but somewhat different from that seen in *Rapetosaurus* (Curry Rogers & Forster, 2004) and *Nemegtosaurus* (Nowinski, 1971), in which the curvature is placed more posteriorly, encompassing alveoli 5–6, or in *Brasilotitan*, in which the dentary is L-shaped, with a right angle between the posterior and anterior regions (Machado et al., 2013).

In medial view, the main body of the dentary is covered by subtle anteroposteriorly oriented ridges, whereas hexagonal interdental plates overlap the alveoli. The centers of these plates are placed at the level of the interalveolar bone, and their anterior and posterior margins extend to the mid-length of the adjacent alveoli. The plates are slightly indented laterally relative to the dentary body, so that a subtle interdental groove is present. Considering their position and hexagonal shape, the interdental plates have their anterior and posterior facets with a dorsoventral orientation, the two dorsal facets with posterodorsal and anterodorsal orientations, and the two ventral facets with posteroventral and anteroventral orientations. The last two facets form the dorsal margin of the interdental foramina, whereas their ventral halves are formed by the dentary body proper. These foramina are subovoid in shape and placed at level of the alveoli. The dimensions of the interdental foramina are similar, whereas the anterior interdental plates are higher than the posterior ones. On the contrary, the interdental foramina of *Brasilotitan* increase posteriorly in size (Machado et al., 2013). The morphology of the interdental plates and foramina of MBC-42-PV is similar to that of *Malawisaurus* (Curry Rogers & Forster, 2004), whereas these structures are more dorsoventrally elongated in *Nemegtosaurus* (Nowinski, 1971).

The poorly preserved lateral surface of the dentary shows some shattered areas. However, a dorsal row of few, broadly spaced nutrient foramina are present, as well as some not-aligned, ventral foramina, as also seen in *Jobaria* (Sereno et al., 1999) and *Malawisaurus* (Curry Rogers & Forster, 2004). The ventral margin of dentary bears two ridges. The most conspicuous lateral ridge forms the lateral/ventral corner of the bone. The medial ridge extends parallel to the lateral ridge, from the posterior margin of the bone as preserved to the level of the sixth alveolus, forming an excavated ventral surface, which corresponds to Meckelian channel. This surface is slightly medially inclined, as the lateral ridge is, for most of its length, more ventrally projected than the medial. At the level of alveoli 4-6, the medial ridge is not more ventrally projected than the lateral ridge, and an almost straight ventral surface is formed. In addition, the ventral surface in this area is sharper due to the confluence of medial and lateral ridges. The anteriormost region of the ventral surface bears a single buttressed ridge. This shows that, as in *Malawisaurus* (Curry Rogers & Forster, 2004) and *Tapuiasaurus* (Zaher et al., 2011), the Meckelian channel of *Maxakalisaurus topai* does not enter the symphyseal articular surface, unlike *Nemegtosaurus* (Nowinski, 1971) in which the channel forms a notch up the ventral third of the symphysis. The symphysis in *Maxakalisaurus topai* is narrow and dorsoventrally high and the anterior tip of the dentary is not dorsoventrally expanded in lateral view, lacking the ventral projection seen in some diplodocoids (Whitlock, Wilson & Lamanna, 2010), the dorsal projection of *Nigersaurus* (Sereno et al., 2007), or the P-shaped symphysis present in *Brasilotitan* (Machado et al., 2013).

**Figure 3:**
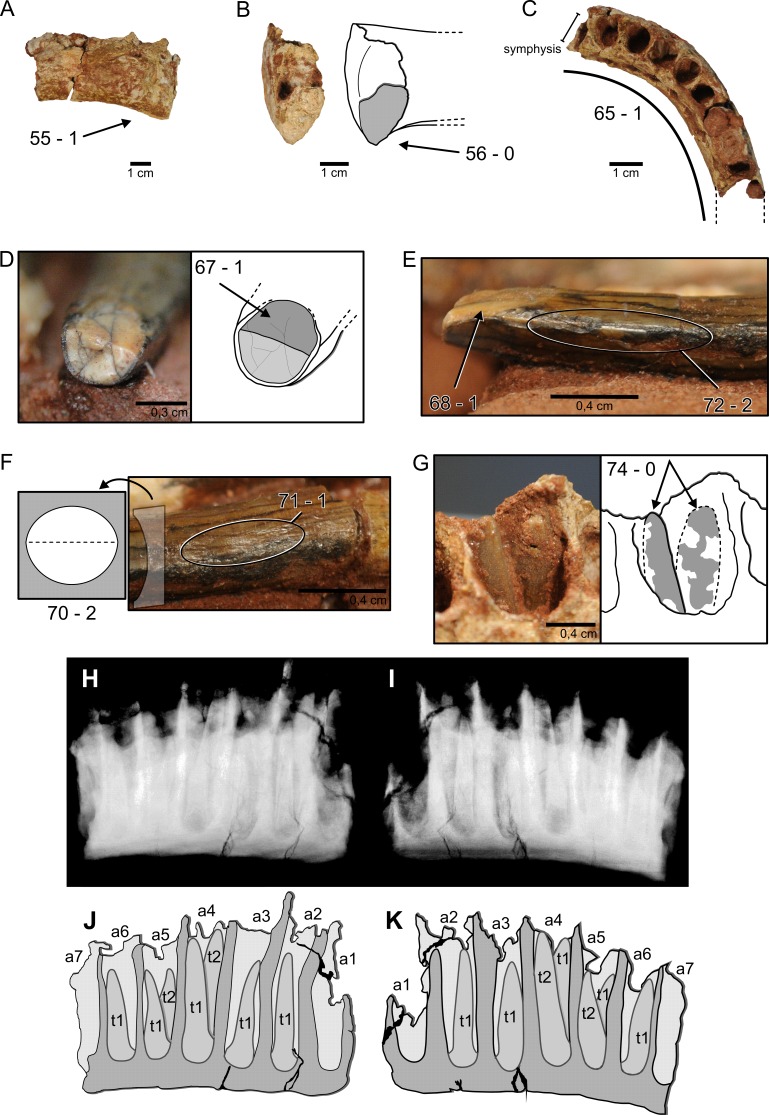
Nine characters newly scored for *M. topai*. (A), Right dentary in labial view. (B), Right dentary in symphyseal view, drawing depicts the outline of its medial portion with preserved part of the symphysis highlighted in gray. (C), Right dentary in dorsal view, dashes outline the missing portions of the ramus. (D), Functional tooth in apical view; light gray indicates broken portion of the tooth, dark gray highlight wear facet. (E), Mesial or distal view of the apical portion of a functional tooth. (F), Mesial or distal view of the mid-crown of the same tooth seen in (D) and (E); drawing indicate the morphology of the tooth cross section. (G), Detail of the fourth alveolus with two replacement teeth (gray shading highlights tooth portions not embedded in the matrix, dashes indicate their possible outline). (H–K), X-ray and draw interpretation of dentary in lingual (H, J) and labial (I, K) views, indicating two replacement teeth per alveoli; a1–a7, alveolus 1–7; t1 and t2, teeth number per alveolus. Numbers refer to characters discussed in the text, followed by the corresponding state score.

No functional tooth is found in the ten preserved dentary alveoli. However, replacement teeth are seen, sometimes two per alveolus, observed on prepared alveoli and X-ray images (Fig. 3G–3H). The first alveolus is not completely preserved, lacking the dorsal part of the mesial, labial, and lingual walls, and does not preserve teeth. Its shape is not clear, although it is longer labiolingally than mesiodistally. The second to fifth alveoli are equivalent in size and the largest of the dentary. In dorsal view, the second to tenth alveoli are oval in shape. The second alveolus bears two replacement teeth, only the apices of which are apparent. Both teeth have a slightly compressed crown, forming carinae. The larger tooth is positioned at the mid-length (mesiodistally) of the alveolus. It is displaced labially relative to the other tooth and bears labiodistally to mesiolingually oriented carinae. The smaller tooth is placed more anteriorly and lingually in the alveolus and has almost labiolingually oriented carinae. This arrangement leaves an empty mesiolingual area, probably occupied by the functional tooth. The third alveolus preserves one replacement tooth, located in its distolabial corner, with almost mesiodistally oriented carinae. This tooth is similar in size (eruption stage) to the smaller tooth of the second, fifth, and seventh alveoli. Two replacement teeth are present in the fourth alveolus. The slightly larger element is mesially displaced, with labiolingually oriented carinae, whereas the smaller tooth is more distally and labially positioned, and has distolabially to mesiolingually oriented carinae. The fifth and sixth alveoli have one replacement tooth each, placed on the distolabial corner and with almost mesiodistally oriented carinae. In dorsal view, the seventh and eight alveoli are filled by unprepared matrix. Yet, the mesial portion of the seventh alveolus is broken, showing two replacement teeth in mesial view. The larger tooth is labially displaced, with mesiodistally oriented carinae. The smaller tooth is positioned in the mesial part of the alveolus and its distal margin is well exposed. It is a slender element, with a basiapical length four times the maximum thickness. The pointed apical region is mesiodistally compressed with labiolingually oriented carinae. On the other hand, the basal region is oval in cross section, with reduced carinae. The ninth alveolus is partially preserved, showing one replacement tooth on its labial region, with almost mesodistally oriented carinae. The tenth alveolus is broken, with no preserved teeth.

The five isolated teeth preserved in MBC-38-PV (Fig. 2) are functional teeth, similar in morphology and relative size with to those of the holotypic maxilla (Kellner et al., 2006). They are arranged subparallel to one another, with no other associated skeletal remains. Such unusual arrangement may result from reorientation during the individual transport of the elements or represent a leftover of their original position on the jaw. The teeth are straight and have high crowns, with winkled and thick enamel. The crown occupies about two thirds of the length of the four better preserved teeth (maximal apicobasal length/crown length in cm are: 4.2/2.3; 4.6/3.3; 3.7/2.6; 4.6/3.4). Their slenderness indices (length of the tooth/maximum mesiodistal width) in cm are, respectively, 4.2, 5.5, 4.7, and 5.6. The transverse section of the crown base is ovoid, whereas the apical region is more compressed, with weak carinae, like the tooth from holotypic maxilla. Serrations are absent from all teeth. Although apically tapering, the tips of the teeth are not pointed, but convex in outline. The apical region of each tooth bears a chisel-shaped wear surface, suggesting a high angled occlusion between teeth.

### New characters scored for *Maxakalisaurus topai*

From the fifteen dentary and teeth characters employed by Santucci & Arruda-Campos (2011) and Zaher et al. (2011) nine are here for the first time scored for *M. topai* (Fig. 3), whereas the remaining six are still indeterminate based on the presently know specimens. The list below describes the character states found in *M. topai*; numbers follow the “Character list” of the Supplemental Information.

*55*- *Dentary, depth of anterior end of ramus: slightly less than that of dentary at midlength (0); 150% minimum depth (1). M. topai* has the anterior end of dentary ramus dorsoventrally expanded in relation to the mid-length of the bone (Fig. 3A). Score = 1.
*56*- *Dentary, anteroventral margin shape: gently rounded (0); sharply projecting triangular process or ‘chin’ (1)*. The anteroventral margin of dentary in *M. topai* lacks a triangular process or ‘chin’; instead, it is slightly rounded (Fig. 3B). Score = 0.
*65*. *Tooth rows, shape of anterior portions: narrowly arched, anterior portion of tooth rows V-shaped (0); broadly arched, anterior portion of tooth rows U-shaped (1); rectangular, tooth-bearing portion of jaw perpendicular to jaw rami (2).* The dentary tooth row of *M. topai* is arched, contrasting with V-shaped or rectangular rows (Fig. 3C). Score = 1.
*67*. *Crown-to-crown occlusion: absent (0); present (1).* Isolated teeth of *M. topai* bear chisel-shaped wear facets (Fig. 3D), indicating the presence of crown-to-crown occlusion. Score = 1.
*68*. *Occlusal pattern: interlocking, V-shaped facets (0); high-angled planar facets (1); low-angled planar facets (2).* The chisel-shaped tooth wear surfaces of *M. topai* (Fig. 3D) are high-angled. Score = 1.
*70*. *Tooth crowns, cross-sectional shape at midcrown: elliptical (0); D-shaped (1); cylindrical (2).* The basal region of *M. topai* teeth is oval, almost circular, in cross section. This morphology is retained at the mid-length of the crown (Fig. 3F), whereas the apical region is more labiolingually compressed, with a suboval cross section. Score = 2.
*71*. *Enamel surface texture: smooth (0); wrinkled (1).* The enamel in *M. topai* teeth is thick and wrinkled (Fig. 3F). Score = 1.
*72*. *Marginal tooth denticles: present (0); absent on posterior edge (1); absent on both anterior and posterior edges (2).* No serrations or denticles are seen in *M. topai* teeth (Fig. 3E). Score = 2.
*74*. *Replacement teeth per alveolus, number: two or fewer (0); more than four (1).* The second, fourth, and seventh alveoli of the *M. topai* dentary bear two replacement teeth (Fig. 3G–3K). Score = 0.

### Phylogenetic analysis

**Figure 4:**
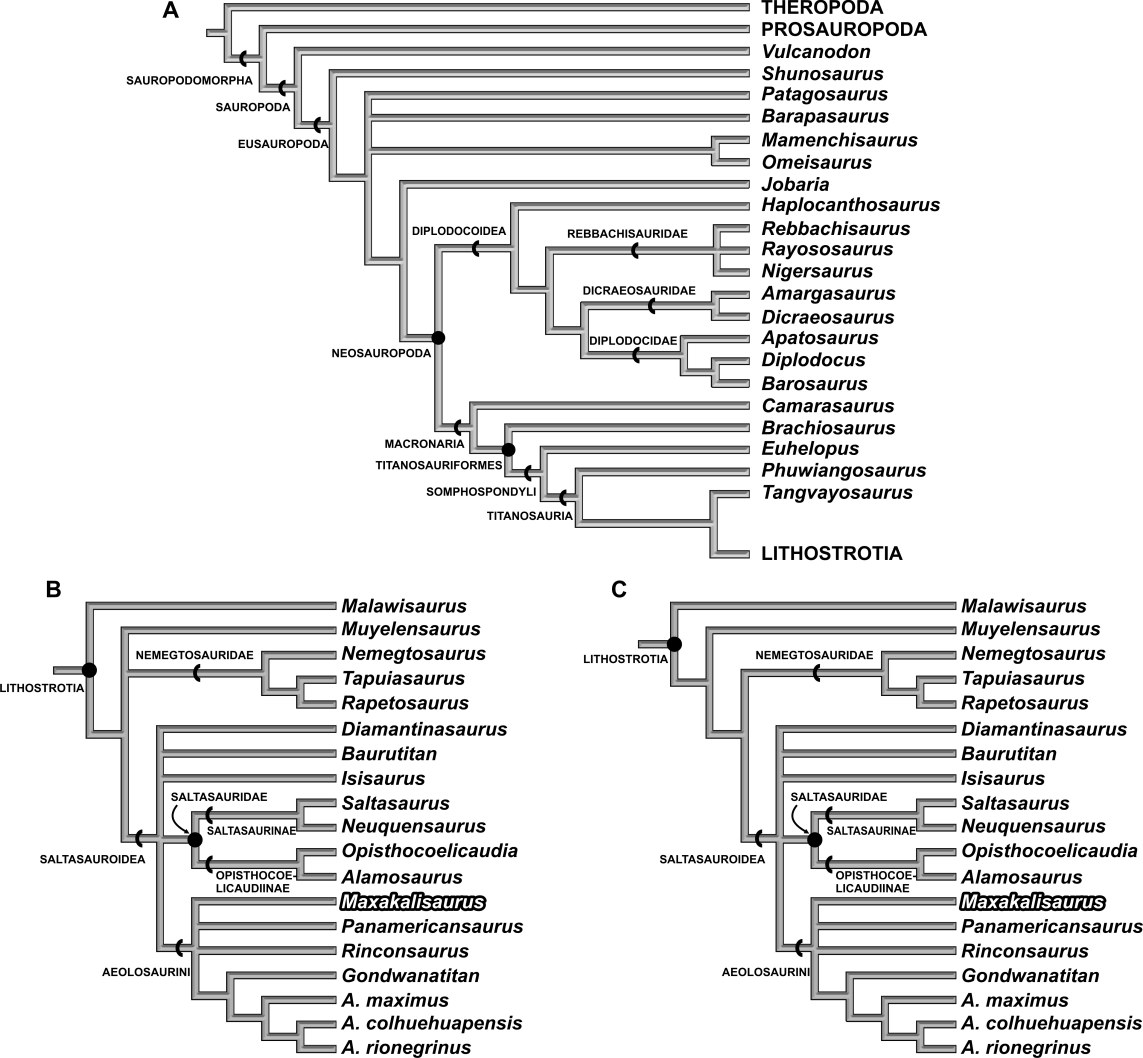
Results of Cladistic Analysis. (A), Strict consensus tree of the two analyses, with the same topology for early sauropodomorphs and macronarians. (B), Strict consensus of 20 most parsimonious tree, with 479 steps, based only on previous scores for *M. topai*. (C), Strict consensus of 8 most parsimonious tree, with 479 steps, based on previous and newly scored characters for *M. topai*.

The first analysis (with *M. topai* score based only on previous works) identified 20 most parsimonious trees (MPTs) with 479 steps (Figs. 4A and 4B). The Aeolosaurini relations proposed by Santucci & Arruda-Campos (2011) were retrieved: *Panamericanus*, *Maxakalisaurus*, and *Rinconsaurus* form a polytomy with the clade composed by *Gondwanatitan* and the species of *Aelosaurus* (*A. maximus*, *A.colhuehuapensis*, and *A. rionegrinus*). Aeolosaurini, Saltasauridae, *Baurutitan*, *Isisaurus*, and *Diamantinasaurus* form an also polytomic clade, here named Saltosauroidea (Figs. 4B and 4C), and branch-based defined as the most inclusive clade to include *Saltasaurus* but not *Nemegtosaurus*. Saltasauroidea plus *Muyelensaurus* and Nemegtosauridae also constitute a polytomic clade, to which *Malawisaurus* is sister taxon as the earliest branching Lithostrotia (Fig. 4B). Within Titanosauria, *Phuwiangosaurus* and *Tangvayosaurus* are basal taxa to Lithostrotia. The relationships of other clades (Eusauropoda, Neosauropoda, Diplodocoidea, Macronaria, Titanosauriformes, and Somphospondyli) are similar to the original analysis of Wilson (2002).

**Figure 5:**
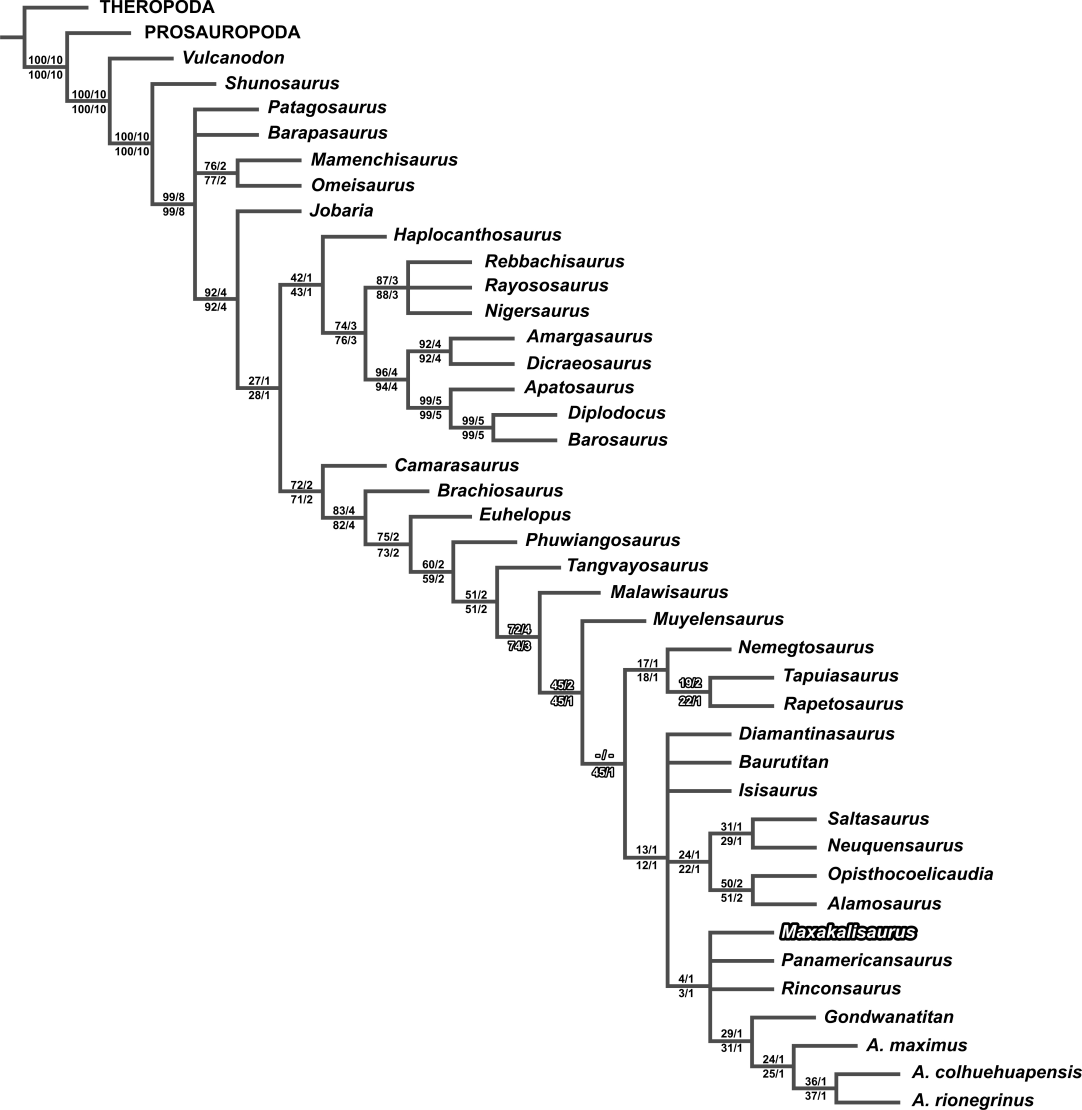
Branch support values plotted on the strict consensus tree of the second analysis (previous and newly scored characters for *M. topai*). Bootstrap values on the left and Bremer support on the right; Above the branch, values for the first cladistics analysis, with *M. topai* based only on previous scores; Below the branch, values for the second cladistics analysis, with *M. topai* based on previous and new scores. Significant differences are highlighted.

The second analysis, including the new scores for *Maxakalisaurus topai*, found eight most parsimonious trees with 479 steps. The topology is almost identical to the first analysis, except for the early Lithostrotia branching. As in the first analysis, *Malawisaurus* is revealed as the earliest splitting member of the group. However, the polytomy up the tree is solved: Nemegtosauridae and Saltasouroidea forming a clade, in which *Muyelensaurus* is sister taxon (Fig. 4C). Although the added information is not related to those taxa, the scoring of *M. topai* may have affected character polarization. Among the synapomorphies found in the second analysis, character 70 supports the affinity of Nemegtosauridae to Saltasauroidea, and has not been previously scored for *M. topai*. In the first analysis, a cylindrical cross section of teeth optimizes as plesiomorphic, with a D-shaped section apomorphic for Eusauropoda, and an oval cross section convergently acquired in Diplodocoidea, *Phuwiangosaurus*, and Nemegtosauridae. The condition in Saltasauroidea is uncertain, because no taxon has been scored for this character, but optimizes as D-shaped through phylogenetic inference. When the oval cross section of *M. topai* is included in the second analysis, character optimization changes, with the cylindrical condition appearing as convergently acquired by Diplodocoidea, *Phuwiangosaurus*, and the clade composed by Nemegtosauridae plus Saltasauroidea. The other eight characters newly scored for *M. topai* do not appear to bear any influence in the results of the second analysis. Statistical comparison between two analysis were performed. The consistency index (CI) and retention index (RI) obtained on first analysis were 0,589 and 0,787, respectively. This is similar to values from second analysis (CI = 0, 589; RI = 0, 788). In addition, branch support tests also indicate similar or equal values in the two analyses for almost all clades (Fig. 5). An exception is observed for early Lithostrotia nodes, as expected due to the distinct topologies of the cladrograms in this area.

### Dentition and tooth replacement in *Maxakalisaurus topai*

Functional teeth in Saltasauroidea, including Aeolosaurini, are known only for *Maxakalisaurus topai* and *Rinconsaurus caudamirus* (Calvo & Riga, 2003). These are, in both cases, straight with suboval cross section (with a slightly more convex labial surface) in the basal portion, bearing chisel shaped wear surface on the apex. These conditions were most probably inherited from their common lithostrotian ancestor, given that similar basic tooth morphology is present in *Malawisaurus*, *Muyelensaurus*, and nemegtosaurids. The minor morphological variations seen within Lithostrotia include the presence of teeth with denticles in the carinae and both chisel (planar high angled) and V-shaped wear surfaces in *Tapuiasaurus* (Zaher et al., 2011), and of more lingually flattened tooth crowns, with a D-shaped cross section, in *Nemegtosaurus* (Wilson, 2005). In addition, some Lithrostotia show variations on tooth position and insertion in the bone, e.g., crown length decrease towards the posterior end of the tooth row in *Tapuiasaurus* and *Malawisaurus*, upper teeth are longer than lower teeth in *Tapuiasaurus*, and both upper and lower teeth have anterolingual and labial curvatures in, respectively, *Malawisaurus* and *Nemegtosaurus* (Nowinski, 1971; Wilson, 2005; Zaher et al., 2011).

Data from the premaxilla of *Diplodocus* and *Camarasaurus* indicate that tooth replacement is labiolingually aligned, with younger teeth lingually positioned in relation to the functional teeth (D’Emic et al., 2013). In dentary of *Maxakalisaurus topai*, functional teeth occupy a mesiolingual position in the alveolous, with two replacement teeth placed distally, distolingually, distolabially, labially, or mesiolabially, depending on the alveolus and on the development degree of the tooth. This is quite similar to the condition seen in the dentary of *Malawisaurus* (Fig. 7B, Gomani, 2015), which could well be plesiomorphic for Titanosauria/Lithostrotia.

## Conclusions

Details of the skull anatomy for Titanosauria are uncommon due to preservation biases, and their classification and phylogeny are grounded on postcranial data, especially for the Saltasauroidea. Of the 15 dentary and teeth characters found in the phylogenetic literature on Sauropods, the new data gathered for *Maxakalisaurus topai* allow the scoring of nine, increasing from 24 to 33 the characters known for that taxon. This scorings did not significantly change previous phylogenetic hypothesis, but the new material helps to better understand the jaw morphology, function, and tooth replacement of *M. topai*, and of Titanosauria as a whole.

##  Supplemental Information

10.7717/peerj.2054/supp-1 Data S1 Phylogenetic Analysis Dataset (A) Phylogenetic Characters (B) Operational Taxonomic Units (C) Phylogenetic Matrix.

## Additional Information and Declarations

### Data Availability

The following information was supplied regarding data availability: The raw data has been supplied as Supplemental Information.
