# Supplementary material for: New lower jaw and teeth referred to Maxakalisaurus topai (Titanosauria: Aeolosaurini) and their implications for the phylogeny of titanosaurid sauropods
Source: PeerJ. 2016 Jun 8;4:e2054. doi: 10.7717/peerj.2054 (PMC4906671; doi:10.7717/peerj.2054)
Supplement: Data S1 — (A) Phylogenetic Characters (B) Operational Taxonomic Units (C) Phylogenetic Matrix. [file peerj-04-2054-s001.pdf]

<u>SUPPLEMENTAL INFORMATION</u>

A- Phylogenetic Characters

The 253 phylogenetic characters are based on: 1-234= characters 1-234 of Wilson (2002); 234-241= characters 235-241 of Santucci & Arruda-Campos (2011); 242-253= characters 235-246 of Zaher *et al*. (2011).

1. Posterolateral processes of premaxilla and lateral processes of maxilla, shape: without midline contact (0); with midline contact forming marked narial depression, subnarial foramen not visible laterally (1).
2. Premaxillary anterior margin, shape: without step (0); with marked step, anterior portion of skull sharply demarcated (1).
3. Maxillary border of external naris, length: short, making up much less than one-fourth narial perimeter (0); long, making up more than one third narial perimeter (1).
4. Preantorbital fenestra: absent (0); present (1).
5. Subnarial foramen and anterior maxillary foramen, position: well distanced from one another (0); separated by narrow bony isthmus (1).
6. Antorbital fenestra, maximum diameter: much shorter than (0) or subequal to (1) orbital maximum diameter.
7. Antorbital fossa: present (0); absent (1).
8. External nares, position: terminal (0); retracted to level of orbit (1); retracted to a position between orbits (2).
9. External nares, maximum diameter: shorter (0) or longer (1) than orbital maximum diameter.

10. Orbital ventral margin, anteroposterior length: broad, with subcircular orbital margin (0); reduced, with acute orbital margin (1).

11. Lacrimal, anterior process: present (0); absent (1).

12. Jugal–ectopterygoid contact: present (0); absent (1).

13. Jugal, contribution to antorbital fenestra: very reduced or absent (0); large, bordering approximately one-third its perimeter (1).

14. Prefrontal, posterior process size: small, not projecting far posterior of frontal–nasal suture (0); elongate, approaching parietal (1).

15. Prefrontal, posterior process shape: flat (0); hooked (1).

16. Postorbital, ventral process shape: transversely narrow (0); broader transversely than anteroposteriorly (1).

17. Postorbital, posterior process: present (0); absent (1).

18. Frontal contribution to supratemporal fossa: present (0); absent (1).

19. Frontals, midline contact (symphysis): sutured (0) or fused (1) in adult individuals.

20. Frontal, anteroposterior length: approximately twice (0) or less than (1) minimum transverse breadth.

21. Parietal occipital process, dorsoventral height: short, less than the diameter of the foramen magnum (0); deep, nearly twice the diameter of the foramen magnum (1).

22. Parietal, contribution to post-temporal fenestra: present (0); absent (1).

23. Postparietal foramen: absent (0); present (1).

24. Parietal, distance separating supratemporal fenestrae: less than (0) or twice (1) the long axis of supratemporal fenestra.

25. Supratemporal fenestra: present (0); absent (1).

26. Supratemporal fenestra, long axis orientation: anteroposterior (0); transverse (1).

27. Supratemporal fenestra, maximum diameter: much longer than (0) or subequal to (1) that of foramen magnum.

28. Supratemporal region, anteroposterior length: temporal bar longer (0) or shorter (1) anteroposteriorly than transversely.

29. Supratemporal fossa, lateral exposure: not visible laterally, obscured by temporal bar (0); visible laterally, temporal bar shifted ventrally (1).

30. Laterotemporal fenestra, anterior extension: posterior to orbit (0); ventral to orbit (1).

31. Squamosal–quadratojugal contact: present (0); absent (1).

32. Quadratojugal, anterior process length: short, anterior process shorter than dorsal process (0); long, anterior process more than twice as long as dorsal process (1).

33. Quadrate fossa: absent (0); present (1).

34. Quadrate fossa, depth: shallow (0); deeply invaginated (1).

35. Quadrate fossa, orientation: posterior (0); posterolateral (1).

36. Palatobasal contact, shape: pterygoid with small facet (0), dorsomedially orientated hook (1), or rocker-like surface (2) for basipterygoid articulation.

37. Pterygoid, transverse flange (i.e. ectopterygoid process) position: posterior of orbit (0); between orbit and antorbital fenestra (1); anterior to antorbital fenestra (2).

38. Pterygoid, quadrate flange size: large, palatobasal and quadrate articulations well separated (0); small, palatobasal and quadrate articulations approach (1).

39. Pterygoid, palatine ramus shape: straight, at level of dorsal margin of quadrate ramus (0); stepped, raised above level of quadrate ramus (1).

40. Palatine, lateral ramus shape: plate-shaped (long maxillary contact) (0); rod-shaped (narrow maxillary contact) (1).

41. Epipterygoid: present (0); absent (1).

42. Vomer, anterior articulation: maxilla (0); premaxilla (1).

43. Supraoccipital, height: twice (0) subequal to or less than (1) height of foramen magnum.

44. Paroccipital process, ventral nonarticular process: absent (0); present (1).

45. Crista prootica, size: rudimentary (0); expanded laterally into ‘dorsolateral process’ (1).

46. Basipterygoid processes, length: short, approximately twice (0) or elongate, at least four times (1) basal diameter.

47. Basipterygoid processes, angle of divergence: approximately 45° (0); less than 30° (1).

48. Basal tubera, anteroposterior depth: approximately half dorsoventral height (0); sheet-like, 20% dorsoventral height (1).

49. Basal tubera, breadth: much broader than (0) or narrower than occipital condyle (1).

50. Basioccipital depression between foramen magnum and basal tubera: absent (0); present (1).

51. Basisphenoid/basipterygoid recess: present (0); absent (1).

52. Basisphenoid–quadrate contact: absent (0); present (1).

53. Basipterygoid processes, orientation: perpendicular to (0) or angled approximately 45° to (1) skull roof.

54. Occipital region of skull, shape: anteroposteriorly deep, paroccipital processes oriented posterolaterally (0); flat, paroccipital processes oriented transversely (1).

55. Dentary, depth of anterior end of ramus: slightly less than that of dentary at midlength (0); 150% minimum depth (1).

56. Dentary, anteroventral margin shape: gently rounded (0); sharply projecting triangular process or ‘chin’ (1).

57. Dentary symphysis, orientation: angled 15° or more anteriorly to (0) or perpendicular to (1) axis of jaw ramus.

58. External mandibular fenestra: present (0); absent (1).

59. Surangular depth: less than twice (0) or more than two and one-half times (1) maximum depth of the angular.

60. Surangular ridge separating adductor and articular fossae: absent (0); present (1).

61. Adductor fossa, medial wall depth: shallow (0); deep, prearticular expanded dorsoventrally (1).

62. Splenial posterior process, position: overlapping angular (0); separating anterior portions of prearticular and angular (1).

63. Splenial posterodorsal process: present, approaching margin of adductor chamber (0); absent (1).

64. Coronoid, size: extending to dorsal margin of jaw (0); reduced, not extending dorsal to splenial (1); absent (2).

65. Tooth rows, shape of anterior portions: narrowly arched, anterior portion of tooth rows V-shaped (0); broadly arched, anterior portion of tooth rows U-shaped (1); rectangular, tooth-bearing portion of jaw perpendicular to jaw rami (2).

66. Tooth rows, length: extending to orbit (0); restricted anterior to orbit (1); restricted anterior to subnarial foramen (2).

67. Crown-to-crown occlusion: absent (0); present (1).

68. Occlusal pattern: interlocking, V-shaped facets (0); high-angled planar facets (1); low-angled planar facets (2).

69. Tooth crowns, orientation: aligned along jaw axis, crowns do not overlap (0); aligned slightly anterolingually, tooth crowns overlap (1).

70. Tooth crowns, cross-sectional shape at midcrown: elliptical (0); D-shaped (1); cylindrical (2).

71. Enamel surface texture: smooth (0); wrinkled (1).

72. Marginal tooth denticles: present (0); absent on posterior edge (1); absent on both anterior and posterior edges (2).

73. Dentary teeth, number: greater than 20 (0); 17 or fewer (1).

74. Replacement teeth per alveolus, number: two or fewer (0); more than four (1).

75. Teeth, orientation: perpendicular (0) or oriented anteriorly relative (1) to jaw margin.

76. Teeth, longitudinal grooves on lingual aspect: absent (0); present (1).

77. Presacral bone texture: solid (0); spongy, with large, open internal cells, 'camellate' (1).

78. Presacral centra, pneumatopores (pleurocoels): absent (0); present (1).

79. Atlantal intercentrum, occipital facet shape: rectangular in lateral view, length of dorsal aspect subequal to that of ventral aspect (0); expanded anteroventrally in lateral view, anteroposterior length of dorsal aspect shorter than that of ventral aspect (1).

80. Cervical vertebrae, number: 9 or fewer (0); 10 (1); 12 (2); 13 (3); 15 or greater (4).

81. Cervical neural arch lamination: well developed, with well defined laminae and coels (0); rudimentary; diapophyseal laminae only feebly developed if present (1).

82. Cervical centra, articular face morphology: amphicoelous (0); opisthocoelous (1).

83. Cervical pneumatopores (pleurocoels), shape: simple, undivided (0); complex, divided by bony septa (1).

84. Anterior cervical centra, height:width ratio: less than 1 (0); approximately 1.25 (1).

85. Anterior cervical neural spines, shape: single (0); bifid (1).

86. Mid-cervical centra, anteroposterior length/ height of posterior face: 2.5–3.0 (0); > 4 (1).

87. Mid-cervical neural arches, height: less than that of posterior centrum face (0); greater than that of posterior centrum face (1).

88. Middle and posterior cervical neural arches, centroprezygapophyseal lamina (cprl), shape: single (0); divided (1).

89. Posterior cervical and anterior dorsal neural spines, shape: single (0); bifid (1).

90. Posterior cervical and anterior dorsal bifid neural spines, median tubercle: absent (0); present (1).

91. Dorsal vertebrae, number: 15 (0); 14 (1); 13 (2); 12 (3); 11 (4); 10 or fewer (5).

92. Dorsal neural spines, breadth: narrower (0) or much broader (1) transversely than anteroposteriorly.

93. Dorsal neural spines, length: approximately twice (0) or approximately four times (1) centrum length.

94. Anterior dorsal centra, articular face shape: amphicoelous (0); opisthocoelous (1).

95. Middle and posterior dorsal neural arches, centropostzygapophyseal lamina (cpol), shape: single (0); divided (1).

96. Middle and posterior dorsal neural arches, anterior centroparapophyseal lamina (acpl): absent (0); present (1).

97. Middle and posterior dorsal neural arches, prezygoparapophyseal lamina (prpl): absent (0); present (1).

98. Middle and posterior dorsal neural arches, posterior centroparapophyseal lamina (pcpl): absent (0); present (1).

99. Middle and posterior dorsal neural arches, spinodiapophyseal lamina (spdl): absent (0); present (1).

100. Middle and posterior dorsal neural arches spinopostzygapophyseal lamina (spol) shape: single (0); divided (1).

101. Middle and posterior dorsal neural arches, spinodiapophyseal lamina (spdl) and spinopostzygapophyseal lamina (spol) contact: absent (0); present (1).

102. Middle and posterior dorsal neural spines, shape: tapering or not flaring distally (0); flared distally, with pendant, triangular lateral processes (1).

103. Middle and posterior dorsal neural arches, ‘infradiapophyseal’ pneumatopore between acdl and pcdl: absent (0); present (1).

104. Middle and posterior dorsal neural spines, orientation: vertical (0); posterior, neural spine summit approaches level of diapophyses (1).

105. Posterior dorsal centra, articular face shape: amphicoelous (0); opisthocoelous (1).

106. Posterior dorsal neural arches, hyposphene– hypantrum articulations: present (0); absent (1).

107. Posterior dorsal neural spines, shape: rectangular through most of length (0); ‘petal’ shaped, expanding transversely through 75% of its length and then tapering (1).

108. Sacral vertebrae, number: 3 or fewer (0); 4 (1); 5 (2); 6 (3).

109. Sacrum, sacricostal yoke: absent (0); present (1).

110. Sacral vertebrae contributing to acetabulum: numbers 1–3 (0); numbers 2–4 (1).

111. Sacral neural spines, length: approximately twice (0) or four times (1) length of centrum.

112. Sacral ribs, dorsoventral length: low, not projecting beyond dorsal margin of ilium (0); high extending beyond dorsal margin of ilium (1).

113. Caudal bone texture: solid (0); spongy, with large internal cells (1).

114. Caudal vertebrae, number: more than 45 (0); 35 or fewer (1).

115. Caudal transverse processes: persist through caudal 20 or more posteriorly (0); disappear by caudal 15 (1); disappear by caudal 10 (2).

116. First caudal centrum, articular face shape: flat (0); procoelous (1); opisthocoelous (2); biconvex (3).

117. First caudal neural arch, coel on lateral aspect of neural spine: absent (0); present (1).

118. Anterior caudal centra (excluding the first), articular face shape: amphiplatyan or platycoelous (0); procoelous (1); opisthocoelous (2).

119. Anterior caudal centra, pneumatopores (pleurocoels): absent (0); present (1).

120. Anterior caudal centra, length: approximately the same (0) or doubling (1) over the first 20 vertebrae.

121. Anterior caudal neural arches, spinoprezygapophyseal lamina (sprl): absent (0); present and extending onto lateral aspect of neural spine (1).

122. Anterior caudal neural arches, spinoprezygapophyseal lamina (sprl)-spinopostzygapophyseal lamina (spol) contact: absent (0); present, forming a prominent lamina on lateral aspect of neural spine (1).

123. Anterior caudal neural arches, prespinal lamina (prsl): absent (0); present (1).

124. Anterior caudal neural arches, postspinal lamina (posl): absent (0); present (1).

125. Anterior caudal neural arches, postspinal fossa: absent (0); present (1).

126. Anterior caudal neural spines, transverse breadth: approximately 50% of (0) or greater than (1) anteroposterior length.

127. Anterior caudal transverse processes, proximal depth: shallow, on centrum only (0); deep, extending from centrum to neural arch (1).

128. Anterior caudal transverse processes, shape: triangular, tapering distally (0); ‘wing-like’, not tapering distally (1).

129. Anterior caudal transverse processes, diapophyseal laminae (acdl, pcdl, prdl, podl): absent (0); present (1).

130. Anterior caudal transverse processes, anterior centrodiapophyseal lamina (acdl), shape: single (0); divided (1).

131. Anterior and middle caudal centra, shape: cylindrical (0); quadrangular, flat ventrally and laterally (1).

132. Anterior and middle caudal centra, ventral longitudinal hollow: absent (0); present (1).

133. Middle caudal neural spines, orientation: angled posterodorsally (0); vertical (1).

134. Middle and posterior caudal centra, anterior articular face shape: flat (0); procoelous (cone shaped) (1); opisthocoelous (2).

135. Posterior caudal centra, shape: cylindrical (0); dorsoventrally flattened, breadth at least twice height (1).

136. Distalmost caudal centra, articular face shape: platycoelous (0); biconvex (1).

137. Distalmost biconvex caudal centra, length-toheight ratio: less than 4 (0); greater than 5 (1).

138. Distalmost biconvex caudal centra, number: 10 or fewer (0); more than 30 (1).

139. Cervical rib, tuberculum–capitulum angle: greater than 90° (0); less than 90°, rib ventrolateral to centrum (1).

140. Cervical ribs, length: much longer than centrum, overlapping as many as three subsequent vertebrae (0); shorter than centrum, little or no overlap (1).

141. Dorsal ribs, proximal pneumatocoels: absent (0); present (1).

142. Anterior dorsal ribs, cross-sectional shape: subcircular (0); plank-like, anteroposterior breadth more than three times mediolateral breadth (1).

143. ‘Forked’ chevrons with anterior and posterior projections: absent (0); present (1).

144. ‘Forked’ chevrons, distribution: distal tail only (0); throughout middle and posterior caudal vertebrae (1).

145. Chevrons, ‘crus’ bridging dorsal margin of haemal canal: present (0); absent (1).

146. Chevron haemal canal, depth: short, approximately 25% (0) or long, approximately 50% (1) chevron length.

147. Chevrons: persisting throughout at least 80% of tail (0); disappearing by caudal 30 (1).

148. Posterior chevrons, distal contact: fused (0); unfused (open) (1).

149. Posture: bipedal (0); columnar, obligately quadrupedal posture (1).

150. Scapular acromion process, size: narrow (0); broad, width more than 150% minimum width of blade (1).

151. Scapular blade, orientation: perpendicular to (0) or forming a 45° angle with (1) coracoid articulation.

152. Scapular blade, shape: acromial edge not expanded (0); rounded expansion on acromial side (1); racquet-shaped (2).

153. Scapular glenoid, orientation: relatively flat or laterally facing (0); strongly bevelled medially (1).

154. Scapular blade, cross-sectional shape at base: flat or rectangular (0); D-shaped (1).

155. Coracoid, proximodistal length: less than (0) or approximately twice (1) length of scapular articulation.

156. Coracoid, anteroventral margin shape: rounded (0); rectangular (1).

157. Coracoid, infraglenoid lip: absent (0); present (1).

158. Sternal plate, shape: oval (0); crescentic (1).

159. Humeral proximolateral corner, shape: rounded (0); square (1).

160. Humeral deltopectoral attachment, development: prominent (0); reduced to a low crest or ridge (1).

161. Humeral deltopectoral crest, shape: relatively narrow throughout length (0); markedly expanded distally (1).

162. Humeral midshaft cross-section, shape: circular (0); elliptical, with long axis orientated transversely (1).

163. Humeral distal condyles, articular surface shape: restricted to distal portion of humerus (0); exposed on anterior portion of humeral shaft (1).

164. Humeral distal condyle, shape: divided (0); flat (1).

165. Ulnar proximal condyle, shape: subtriangular (0); triradiate, with deep radial fossa (1).

166. Ulnar proximal condylar processes, relative lengths: subequal (0); unequal, anterior arm longer (1).

167. Ulnar olecranon process, development: prominent, projecting above proximal articulation (0); rudimentary, level with proximal articulation (1).

168. Ulna, length-to-proximal breadth ratio: gracile (0); stout (1).

169. Radial distal condyle, shape: round (0); subrectangular, flattened posteriorly and articulating in front of ulna (1).

170. Radius, distal breadth: slightly larger than (0) or approximately twice (1) midshaft breadth.

171. Radius, distal condyle orientation: perpendicular to (0) or bevelled approximately 20° proximolaterally (1) relative to long axis of shaft.

172. Humerus-to-femur ratio: less than 0.60 (0); 0.60 or more (1).

173. Carpal bones, number: 3 or more (0); 2 or fewer (1).

174. Carpal bones, shape: round (0); block-shaped, with flattened proximal and distal surfaces (1).

175. Metacarpus, shape: spreading (0); bound, with subparallel shafts and articular surfaces that extend half their length (1).

176. Metacarpals, shape of proximal surface in articulation: gently curving, forming a 90° arc (0); Ushaped, subtending a 270° arc (1).

177. Longest metacarpal-to-radius ratio: close to 0.3 (0); 0.45 or more (1).

178. Metacarpal I, length: shorter than (0) or longer than (1) metacarpal IV.

179. Metacarpal I, distal condyle shape: divided (0); undivided (1).

180. Metacarpal I distal condyle, transverse axis orientation: bevelled approximately 20° proximodistally (0) or perpendicular (1) with respect to axis of shaft.

181. Manual digits II and III, phalangeal number: 2- 3-4-3-2 or more (0); reduced, 2-2-2-2-2 or less (1); absent or unossified (2).

182. Manual phalanx I.1, shape: rectangular (0); wedge-shaped (1).

183. Manual nonungual phalanges, shape: longer proximodistally than broad transversely (0); broader transversely than long proximodistally (1).

184. Pelvis, anterior breadth: narrow, ilia longer anteroposteriorly than distance separating preacetabular processes (0); broad, distance between preacetabular processes exceeds anteroposterior length of ilia (1).

185. Ilium, ischial peduncle size: large, prominent (0); low, rounded (1).

186. Iliac blade dorsal margin, shape: flat (0); semicircular (1).

187. Iliac preacetabular process, orientation: anterolateral to (0) or perpendicular to (1) body axis.

188. Iliac preacetabular process, shape: pointed, arching ventrally (0); semicircular, with posteroventral excursion of cartilage cap (1).

189. Pubis, ambiens process development: small, confluent with (0) or prominent, projecting anteriorly from (1) anterior margin of pubis.

190. Pubic apron, shape: flat (straight symphysis) (0); canted anteromedially (gentle S-shaped symphysis) (1).

191. Puboischial contact, length: approximately one third (0) or one-half (1) total length of pubis.

192. Ischial blade, length: much shorter than (0) or equal to or longer than (1) pubic blade.

193. Ischial blade, shape: emarginate distal to pubic peduncle (0); no emargination distal to pubic peduncle (1).

194. Ischial distal shaft, shape: triangular, depth of ischial shaft increases medially (0); bladelike, medial and lateral depths subequal (1).

195. Ischial distal shafts, cross-sectional shape: Vshaped, forming an angle of nearly 50° with each other (0); flat, nearly coplanar (1).

196. Femoral fourth trochanter, development: prominent (0); reduced to crest or ridge (1).

197. Femoral lesser trochanter: present (0); absent (1).

198. Femoral midshaft, transverse diameter: subequal to (0), 125–150%, or (1) at least 185% (2) anteroposterior diameter.

199. Femoral shaft, lateral margin shape: straight (0); proximal one-third deflected medially (1).

200. Femoral distal condyles, relative transverse breadth: subequal (0); tibial much broader than fibular (1).

201. Femoral distal condyles, orientation: perpendicular or slightly bevelled dorsolaterally (0) or bevelled dorsomedially approximately 10° (1) relative to femoral shaft.

202. Femoral distal condyles, articular surface shape: restricted to distal portion of femur (0); expanded onto anterior portion of femoral shaft (1).

203. Tibial proximal condyle, shape: narrow, long axis anteroposterior (0); expanded transversely, condyle subcircular (1).

204. Tibial cnemial crest, orientation: projecting anteriorly (0) or laterally (1).

205. Tibia, distal breadth: approximately 125% (0) or more than twice (1) midshaft breadth.

206. Tibial distal posteroventral process, size: broad transversely, covering posterior fossa of astragalus (0); shortened transversely, posterior fossa of astragalus visible posteriorly (1).

207. Fibula, proximal tibial scar, development: not well-marked (0); well-marked and deepening anteriorly (1).

208. Fibula, lateral trochanter: absent (0); present (1).

209. Fibular distal condyle, size: subequal to shaft (0); expanded transversely, more than twice midshaft breadth (1).

210. Astragalus, shape: rectangular (0); wedgeshaped, with reduced anteromedial corner (1).

211. Astragalus, foramina at base of ascending process: present (0); absent (1).

212. Astragalus, ascending process length: limited to anterior two-thirds of astragalus (0); extending to posterior margin of astragalus (1).

213. Astragalus, posterior fossa shape: undivided (0); divided by vertical crest (1).

214. Astragalus, transverse length: 50% more than (0) or subequal to (1) proximodistal height.

215. Calcaneum: present (0); absent or unossified (1).

216. Distal tarsals 3 and 4: present (0); absent or unossified (1).

217. Metatarsus, posture: bound (0); spreading (1).

218. Metatarsal I proximal condyle, transverse axis orientation: perpendicular to (0) or angled ventromedially approximately 15° to (1) axis of shaft.

219. Metatarsal I distal condyle, transverse axis orientation: perpendicular to (0) or angled dorsomedially to (1) axis of shaft.

220. Metatarsal I distal condyle, posterolateral projection: absent (0); present (1).

221. Metatarsal I, minimum shaft width: less than (0) or greater than (1) that of metatarsals II–IV.

222. Metatarsal I and V proximal condyle, size: smaller than (0) or subequal to (1) those of metatarsals II and IV.

223. Metatarsal III length: more than 30% (0) or less than 25% (1) that of tibia.

224. Metatarsals III and IV, minimum transverse shaft diameters: subequal to (0) or less than 65% (1) that of metatarsals I or II (1).

225. Metatarsal V, length: shorter than (0) or at least 70% (1) length of metatarsal IV.

226. Pedal nonungual phalanges, shape: longer proximodistally than broad transversely (0); broader transversely than long proximodistally (1).

227. Pedal digits II–IV, penultimate phalanges, development: subequal in size to more proximal phalanges (0); rudimentary or absent (1).

228. Pedal unguals, orientation: aligned with (0) or deflected lateral to (1) digit axis.

229. Pedal digit I ungual, length relative to pedal digit II ungual: subequal (0); 25% larger than that of digit II (1).

230. Pedal digit I ungual, length: shorter (0) or longer (1) than metatarsal I.

231. Pedal ungual I, shape: broader transversely than dorsoventrally (0); sickle-shaped, much deeper dorsoventrally than broad transversely (1).

232. Pedal ungual II–III, shape: broader transversely than dorsoventrally (0); sickle-shaped, much deeper dorsoventrally than broad transversely (1).

233. Pedal digit IV ungual, development: subequal in size to unguals of pedal digits II and III (0); rudimentary or absent (1).

234. Osteoderms: absent (0); present (1).

235. Apex of the convexity of the posterior articulation on anterior and middle caudal vertebrae: (0) concentrical or slightly displaced above the centrum midline; (1) strongly displaced upward, so that the apex of the posterior articulation is flushed to the level of the dorsal margin of the centrum.

236. Anterior margin of the anterior caudal vertebrae: (0) vertical; (1) strongly inclined forward.

237. Articular facets of the prezygapophyses on anterior and middle caudal vertebrae: (0) normal, not expanded; (1) wide, with a dorsal and a ventral expansion or protuberance.

238. Prezygapophyses curved downward on anteriormost caudal vertebrae: (0) absent; (1) present.

239. Postzygapophyses located on the anterior half of the centrum on anterior and middle caudal vertebrae: (0) absent; (1) present.

240. Haemal arches with double articular facets set in a concave posterodorsal surface: (0) absent; (1) present.

241. Length proportions of the prezygapophyses with respect to the centrum length in middle caudal vertebrae: (0) less than 40%; (1) between 40-50%; (2) more than 50% .

242. maxilla, jugal process: (0) robust, broadly contacting the jugal; (1) tapering posteriorly

243. Ventral edge of anterior surface of the quadratojugal: (0) straigth, not expanded ventrally; (1) concave due to a ventral expansion of the anterior region.

244. Lacrimal, anterior process: (0) short, less than 50% of the length of the ventral process; (1) long, at least 75% of the length of the ventral process.

245. Prefrontal, anterior process: (0) absent; (1) present.

246. Prefrontal, width at the level of the frontal contact: (0) large, equal or longer that the anteroposterior length of the prefrontal; (1) narrow, less than half the anteroposterior length of the prefrontal.

247. Pterygoid, sutural contact with ectopterygoid: (0) on the lateral surface of the ectopterygoid; (1) on the medial surface of the ectopterygoid.

248. Pterygoid, sutural contact with ectopterygoid: (0) broad, along the medial or lateral surface; (1) narrow, restricted to the anterior tip of the ectopterygoid.

249. Basisphenoid, sagital ridge between basipterygoid processes: (0) absent; (1) present.

250. Squamosal, participation in supratemporal fenestra: (0) present; (1) present

251. Maxilla, foramen anterior to the preantorbital fenestra: (0) absent; (1) present

252. Postorbital, posterior margin articulating with the squamosal: (0) with tapering posterior process; (1) with a deep posterior process.

253. Preantorbital fenestra, deep and large anteroposteriorly oriented fossa: (0) opening directly on the lateral side of the maxilla or recessed in a small and shallow fossa; (1) recessed into a deep and large anteroposteriorly oriented fossa.

B- Operational Taxonomic Units

The numbers [1], [2] and [3] indicate original scores and taxa on analysis of Wilson (2002), Santucci & Arruda-Campos (2011), and Zaher *et al*. (2011), respectively;

Theropoda (outgroup) [1], [2], [3]

Prosauropoda [1], [2], [3]

Vulcanodon [1], [2], [3]

Barapasaurus [1], [2], [3]

Omeisaurus [1], [2], [3]

Shunosaurus [1], [2], [3]

Patagosaurus [1], [2], [3]

Mamenchisaurus [1], [2], [3]

Apatosaurus [1], [2], [3]

Barosaurus [1], [2], [3]

Brachiosaurus [1], [2], [3]

Camarasaurus [1], [2], [3]

Dicraeosaurus [1], [2], [3]

Diplodocus [1], [2], [3]

Haplocanthosaurus [1], [2], [3]

Amargasaurus [1], [2], [3]

Euhelopus [1], [2], [3]

Jobaria [1], [2], [3]

Malawisaurus [1], [2], [3]

Nigersaurus [1], [2], [3]

Rayososaurus [1], [2], [3]

Rebbachisaurus [1], [2], [3]

Alamosaurus [1], [2], [3]

Nemegtosaurus [1], [2], [3]

Neuquensaurus [1], [2], [3]

Opisthocoelicaudia [1], [2], [3]

Rapetosaurus [1], [2], [3]

Saltasaurus [1], [2], [3]

Isisaurus colberti [1], [2], [3]

Baurutitan britoi [2]

Maxakalisaurus topai [2]

Aeolosaurus maximus [2]

Aeolosaurus rionegrinus [2]

Aeolosaurus colhuehuapensis [2]

Gondwanatitan faustoi [2]

Rinconsaurus caudamirus [2]

Muyelensaurus pecheni [2]

Panamericansaurus schroederi [2]

Tapuiasaurus [3]

Phuwiangosaurus [3]

Tangvayosaurus [3]

Diamantinasaurus [3]

C- Phylogenetic Matrix

'THEROPODA'

0000000000000000000000000000000??00000000000000000000000000000?0000
0000000010?000000?000000000000000000?0000000000000000000000000??00000?00
0001000000000001100?000000000000000000000000000000000000000000000000?
?0000000000000?000000001100000?0?

'PROSAUROPODA'

0000000000000000000000000000000??00000000000000000000000000000?0000
0000000110?000000?100000000000000000?0000000000000000000000000??00000?00
0000000000000001000?000000000001000000000000000000000000000000000000000
00000000000000?000000001100000?0?

'Vulcanodon'

????????????????0??0???
?????????????????????????1????0??0?00???????100001????????????00??10?????????10

10?11101001????0???????1???0001010101010???000?00001000010?100100100?011000?00
0000????????????
'Barapasaurus’
??1?0????00??
01???0100??10101101110100001100?0??0?00???????1000000000?????01?00?011000000
0?0101011110100????????????011000101010111010001011111?1?10???010???????1??11
?0?000000????????????
'Omeisaurus’
11100011011?000?01011000010111?11?0?1??11100?0?0?0?00110000?1???111011111?
0001040111010?0?3101?110111000000021101?0100000000000010000?000???100?110000
11000?00000101011010100101000000111?110001010101?1000?01011??0101001111111
11111111110?000000????????0?0?
'Shunosaurus’
011000110110000000001??0000011101100010011000000000?000110000?101?11101112
0?00000311?100100?210000?00000000?010?0000100000000000010000?000???0?001110
1010000000000101011010100101000001101011000101 0?01110100?101?10010?0011110
1110111?111110?000000000????00?0?
'Patagosaurus’
1?100?11????????0????????????????????????1??????????????0?????????1?10111???0001?
?011?00100??10101101??0100002??0??????00???????1???0?000???1?00??00??110?0?00
0?0101111110100?????????????1100010101011101000001??????????101011???????????
0?00000?????????????
'Mamenchisaurus’
???????????????????????????????110????????????????????10000?10111?1?1111000011

?401?101011031010?1?1?10?00002?????01101000000010000?000???10001100?01?????
????010101111101001????????????1100????0101110100110 1?1101110?11?????1?11?1??1
??00000000??????????0?

'Apatosaurus’

00111112011?111?010111?101011111100020?1?000010000011?????????221202121?
??011401101011115101111111100000021?110011000011110111110000011111 0011001
0110011000001010110101001111100001111110011010001110100110111011110111111
1111?1111?1110000000000000??00?0?

'Barosaurus’

??01??0
11?111?11?101????111000000?????0?11011111110111111110011????01?00?01????????
0??????????????????????????????11???0?000
000????????????

'Brachiosaurus’

1111001111110001010110000101111111001011110000000110011001111112111001 12
10001103011001100?3101011111110010021 1010?100000001100100000000???1011??10
??110101000001010111101001111111110111101011101111111001101111111 1001111
0111?11?1??11?0?0000000000000?0000

'Camarasaurus’

111100111111000101011001010111001101101111000000001001100111111111101112
1000010201101010103101011011110010021101001000000011011000000000??100?101
0101101010000010101111010011111110011111100011101111101001101110111100111
1011111111111110?000000000000000000

'Dicraeosaurus’

00?11?????0??00?01111111011111?????1?0??1000111010101110???????2212021?0001120110101011311111001110000012 1?1?0?110000101101110000000 11?11001?00??110001000?010101110???1???????????111001101000111010011011101111 0??111111?11????11??0?000000????????0???

'Diplodocus’

00111112011111110101110101011111100020111000010000101111010110 1?22120212111001140110111111510111111110000002111100110111111101111111100111110011001011000100000101011110100 1???????????111001101000111010111 01?101111001111111111111?1111000000000000001100000

'Haplocanthosaurus’

????????????????0????????????????????????????????0???????????????????????????01?3011000100?2101011011110000021101 0?1000000011001000000 0????1100??10??100001000???????????????????????????1110001010111110100????????????????????????????????0?00000?????????????

'Amargasaurus’

??????????1??00101111?1101111?????????????00111011?011?????????????????????001301??1010105111????1??0000?12??1????0?????????????????0???????0??????1??0?1????0101011010?001?????????????1100???????1110?00???????????????????????????????0??0?000????????0???

'Euhelopus’

01110?11??1????10???????0???????11???011??????????????1001??????1110111 21?1011?4011101101121010111011001?03??01??????????????????????????1011??????110010

000?110101???????????????????111??01??0111111001101?10111?00?11101?11?1?1?11
??0???????????????0000
'Jobaria'
1111001111100010101100001011101110?10?11?000??000?001000??????111011100
00001?3011000100?31010111111000002110100100000001100100000000???10001?001
01101010000010101111010010111000011111100010101111101001101110111100111100
11111????????0?000000??????????00
'Malawisaurus'
11?????1??1??100???????1?1??11210?011?
?1100011?0??10101?01?1101110???????1??10?001110100001000???101?0?11??1?????0
0011101011000100???1??111?????????????111?111????11110???????1??????????????1??
11000001???????0????
'Nigersaurus'
0011??1100???0011?001???1????1?1110???????0001?00??01110?100????22110212010
101??0110001?0??1100???????1020?0
???
???00???????????
'Rayososaurus'
?????????01??0011?00110?1???110?110??0????10010101101??????????????1??212???1
01??011000100??1?10???1110000?1?????001000000001101100000000011?1?????1???1102
0?0001010001111010 01?????????????11??01010111110?00?10??1?1???0??10001111?????
????0?00000?????????????
'Rebbachisaurus'
??1???

???????0??11??111111000011???110201?????1?
0??????????????????????????????011??????????????????????????????????????0?????????
??????????
'Alamosaurus'
??11??1
100001?0??1010110101101110??????1231100001101100001010???????0?111111101111
?11111101100111???1111112??????????0111??????????????????????????????????????00
000000????????????
'Nemegtosaurus'
01?1?011?10100010101010101010101111211111?11000100010110111??01?111?02121
000???
??
????01?10??01111
'Neuquensaurus'
???11??0
10?001?0??101????1?1?0111031?0?1??3010?0011?110000101110??????????111010111
11111101101111?????????????111101?01111121111111?1011101??1110?????1???????
100000?0????????????
'Opisthocoelicaudia'
??1???
??????0104101011110110111031?0??1221200001101100001020100??110?11111100111
11111111011011111??1111112??1111?011011111211101111101110111111011111110
11110?000000????????????

'Rapetosaurus'

0011?111?10?000?0001010101010101010?1112111???11?0100?110100111????1111021210
0011??110?011?0??10101?01011011103?????????1??0???????00???1????1?????????1110
??10011101101?00110?????1?1????1110101101111?1101???????????????????????????????
???101000??1?1110110000

'Saltasaurus'

?????????????00??101?00101????????????????01000101???1??????????????????????11
??011000110??10101?1101101110312011????10?00111110000101110?1?????11?011101
011111111011011111?????????????1111101101111211111111110?????????????????????
???10000000????????????

'Aeolosaurus_maximus'

??1???0
??????????????????0????11??????0????10?000000100?01211????0110?11?01???????????
?1??????????????????????????????1?112110???????????????????????????????????0101112
????????????

'Aeolosaurus_rionegrinus'

??
?????????????????????????????????????11?00?1??100?012?????????0?10??11?0?0?????1?
1111?01????????10???????????????1?1??1111112
????????????

'Aeolosaurus_colhuehuapensis'

??
???????????????????????????????0?1??110001???100?0121????????0?1??01????????????

???0111112??
??????????
'Gondwanatitan_faustoi'
??11???
?????????101???0???0011103????0??0?10000?1?0100?01211?????1?0?1???1???1?????1
10111?????????????????????1??????11???????001????????????????????????????01001
?2????????????
'Muyelensaurus_pecheni'
?????????????????0??00?00?????????????????01????0??????????????????1?1??????11??
110??11?0??101011010000?110??????????10?0011?0??????01????11????????1100?????1
11????????????????????????????011011111?11???1?????1????????????????????????0100
0?1????????????
'Tapuiasaurus'
00?1?111?10?100101?10??10101110111??1111??11?0?00?110?001?0??1??111102121?
0011??01???11????10?0110101??011??????????????????????????????????11??????1?????
10?1???1??110011??????1??????????????????1?2?????????1?????????????????1110?111?
???????111111111111
'Phuwiangosaurus'
???????????????10101????0?????0?110???????10001101000 1????????????110212???00
1??0110011010?1010111101100100?1?0100?00000?0110010000000010011110?101?110
111???10101011?011101???????????1110101111111121101110111 1????????111?????1??
?????0???????????????01?0?
'Tangvayosaurus'
??1???

1????????????01?11???0?10??????00?00000?0??1110000000100???10?111?1??????????
?????????????????????????????0110111121?0?110111011100???1001111?111001110??
?????????????????
'Diamantinasaurus'
???11???
??11??????111010???11111
111101???1??11?1111111111101101111210011111111111100?????????????????????????
??????????????
'Baurutitan_britoi'
??
?????????????????????????????????131100001100100?0101????????0?11???????????????
??0000001??
??????????
'Isisaurus_colberti’
???1??1
10000100??101011010100?1103120??????10?001101100001010???1??10?11?0101010??
??1101111101???????????????1111101101111???????????????????????????????????????0
00000?????????????
'Rinconsaurus_caudamirus'
??????????????0??2???0?1??
010?00110??10???????????110??????????10????????0??00011???????0?1???1????0?1?1?
101?1??000000
1????????????

'Panamericansaurus_schroederi'

??
????????????????????????????????????100???????00?0?21????????0?1?????????????110?
?1???00100?1???
?????????

'Maxakalisaurus_original'

???1
1????1?0????????????????1???????????10????????00?01?11???1???0?11???????????1?10
???100???1???
??????????

'Maxakalisaurus_new'

???10????????1?11?212?0??????
11????1?0????????????????1???????????10????????00?01?11???1???0?11???????????1?1
0???100???1??
???????????

D- References

Santucci RM, Arruda-Campos AC. 2011. A new sauropod (Macronaria, Titanosauria) from the Adamantina Formation, Bauru Group, Upper Cretaceous of Brazil and the phylogenetic relationships of Aeolosaurini. *Zootaxa* 3085: 1-33.

Wilson JA. 2002. Sauropod dinosaur phylogeny: critique and cladistica analysis. *Zoological Journal of the Linnean Society* 136 (2): 217–276. doi:10.1046/j.1096-3642.2002.00029.x

Zaher H, Pol D, Carvalho AB, Nascimento PM, Riccomini C, Larson P, Juarez-Valieri R, Pires-Domingues R, Da Silva NJ, Campos DA. 2011. A Complete Skull of an Early Cretaceous

Sauropod and the Evolution of Advanced Titanosaurians. *PLoS ONE* 6(2): e16663.
doi:10.1371/journal.pone.0016663
